# Rostering in Air Traffic Control: A Narrative Review

**DOI:** 10.3390/ijerph19084625

**Published:** 2022-04-12

**Authors:** Michela Terenzi, Orlando Ricciardi, Francesco Di Nocera

**Affiliations:** 1 Deep Blue SRL, 00193 Rome, Italy; michela.terenzi@dblue.it; 2 Department of Psychology, Sapienza University of Rome, 00185 Rome, Italy; orlando.ricciardi@uniroma1.it; 3 Department of Planning, Design, and Technology of Architecture, Sapienza University of Rome, 00196 Rome, Italy

**Keywords:** shift workers, shift patterns, night shifts, night workers, air traffic controllers, circadian rhythm

## Abstract

Most Air Traffic Controllers (ATCOs) must cover uninterrupted work shifts for 24 h a day, seven days a week. The proper planning of a shift schedule requires consideration of at least three elements: the specific characteristics of the controller task, the physiological needs of the operator, and the definition of rest periods within rostering. We reviewed the literature for providing comprehensive guidance on the main requirements for the construction of a shift schedule for ATCOs. Our considerations are organized according to a rationale reflecting the most important criteria for the construction of the schedule: namely, the organization of rest periods conceptualized as intervals between cycles of shifts, intervals between individual shifts, and breaks within the shift. The suggested parameters could be used to construct shift schedules within a variation margin that depends on individual contexts of application.

## 1. Introduction

Most Air Traffic Controllers (ATCOs) must cover uninterrupted work shifts for 24 h a day, seven days a week. In these operational contexts, fatigue can adversely affect performance by increasing response time and the number of errors [1,2,3]. Additionally, shift workers can suffer consequences for sleep quality and job satisfaction [4,5,6]. Properly organized shifts can mitigate the negative consequences of fatigue accumulation. One of the critical elements of a work shift is scheduling rest periods, intended as the time intervals between consecutive turns and as breaks during the single period of service [7,8,9].

Three elements contribute to creating a fit and safe shift planning. The first is related to the specific characteristics of controller tasks, which usually reflect different levels of complexity and, therefore, require varying amounts of effort. The second element is the physiological need of the operator, which varies according to individual differences. Task characteristics and physiological needs greatly affect the third element, which is the organization of rest periods.

### 1.1. Task Characteristics

Based on different information sources (e.g., radar screen, paper, or electronic flight strips), air traffic controllers must manage a complex, dynamic, and time-constrained traffic situation to identify and solve potential conflicts and optimize the flight paths without impacting safety. Therefore, they must perceive, comprehend, and anticipate multiple characteristics and flight paths of many aircraft while new incoming aircraft create new traffic geometry for evaluation. ATCOs’ diagnosis, decision making, and action implementation are based on these insights into current and anticipated structures of the changing situation. These types of tasks may require maintaining a high level of attention and engagement for an extended time and for specific actions to discriminate against the presence of target stimuli, typical features of a vigilance task. Studies conducted on performance in supervisory tasks indicate a decline in performance after a short (ranging from 20–30 min) [10,11] or more extended period of time (2 h) [12,13,14,15]. These results suggest the necessity of planning breaks during the shift to avoid decreased performance. However, variability in sizes and densities of airspace and time of day, together with specific requirements for each Air Navigation Service Provider (ANSP), make it impossible to standardize the frequency and duration of a break.

### 1.2. Physiological Needs

The most influential variable inherent to individual characteristics is represented by changes in biological rhythms that occur during work over a 24-h period [16,17]. The alteration in biological rhythms, especially during night shifts, is one of the main reasons to implement a Fatigue Risk Management System (FRMS), as ICAO requires from 2020. The report published by the International Civil Aviation Organization (ICAO), the Civil Air Navigation Services Organization (CANSO), and the International Federation of Air Traffic Controllers’ Associations (IFATCA) provides a comprehensive description of the influence of biological rhythms in the air traffic management (ATM) industry [18], with particular emphasis on work in the nighttime. When the work shift involves nighttime hours, there is an impact on the quality and quantity of sleep, producing a psychophysiological feeling of fatigue. In a study involving 13 ATCOs, controllers reported a higher level of fatigue on the night shift than on the morning and evening shifts, despite fewer aircraft to control [19]. The level of fatigue during a real work shift was reported to be lower in the evening shift, although it was the shift with the highest number of aircraft. The study analyzed results obtained in a real context and not in simulation, making the results even more interesting and highlighting how other factors, in addition to the task load, affect the perception of fatigue, such as sleepiness. This study suggests that task load is not a major determinant of fatigue level. However, task load can still interact with fatigue, increasing the probability of making errors, as reported in a study on 57 ATCOs [20].

### 1.3. Organization of Rest Periods

The third element is the focus of this review and is the management of breaks during shift work. Although several studies address shift workers (see below), at least two problems have prevented a definition of guidelines for the design of ATCOs’ shift schedules. The first problem is related to the generalization of the shift worker figure; numerous studies summarize information obtained on different professional categories, such as chemical employees [21], health workers [22,23,24], workers in nuclear power plants [25], and mine workers [26]. Although these workers share some aspects of 24-h rostering, such as working during night hours and loss of sleep, essential differences in activities carried out prevent a real generalization of the results. On the other hand, some shift work characteristics cut across occupations, which is why certain studies were included in this review. Sleep research results conducted on the general population, for example, cannot be excluded from the analysis because this basic and applied research can strongly contribute to the understanding of how humans deal with shifts. In conclusion, the generalization of research findings involving other populations should be considered carefully but not be excluded from the analysis. The second problem is the lack of an organic analysis of the roster variables, which are often analyzed independently. For example, some studies have focused on the effectiveness of breaks [27,28,29], while others have analyzed the role of the length of the work shift [30,31,32]. Still, others have examined the direction and speed of shift rotation [33] or the influence of working during night hours [34,35].

The objective of this review is to integrate those studies with the aim to provide comprehensive guidance on the main requirements for constructing a shift schedule for ATCOs.

## 2. Methods

An extensive though not systematic search conducted on major databases (i.e., Pubmed, PsychINFO, Web of Science, and Scopus) allowed the identification of relevant studies for this literature review, combining the keywords “shift working” or “shift rotation” and “air traffic controllers”. Only English written peer-reviewed articles were selected. Considering the narrative nature of this review, we considered any information we came across to be valuable and no interval dates were set. The articles were first screened for relevance by reading the title alone and then filtered by reading the abstract. Ninety-six full-text articles were included in this analysis.

## 3. Results

The results are organized according to a rationale reflecting the main requirements for the construction of the schedule: namely, the organization of rest periods conceptualized as intervals between cycles of shifts, intervals between individual shifts, and breaks within the shift.

### 3.1. Shift Patterns Used by Leading Air Nation Service Providers

The shift scheme is one of the most critical features in defining rest periods within a shift cycle. Relevant information on this issue comes from a 2006 report by EUROCONTROL [36], the international organization working to achieve safe and seamless air traffic management across Europe. This document aimed at identifying best practices to help define solutions for the management of shift work in air traffic management (ATM) and other sectors. The study resulted in some recommendations to facilitate the planning and management of flexible working practices to improve safety and productivity. Another major objective of the EUROCONTROL study was also to compare shift workers’ management practices of 10 ANSPs from the European Union and three International ANSPs. European Union (EU) suppliers included Austrocontrol (Austria), AENA (Spain), ANS (Czech Republic), Avinor (Norway), DGAC (France), DFS (Germany), LFV (Sweden), Maastricht (EUROCONTROL), NATS (UK), and Skyguide (Switzerland). The comparison revealed that the schedules’ features vary from country to country and depend on the presence of the roster organized by a team or by an individual. The prevailing schemes include 4 working days and 2 rest days (4/2), 5 working days and 2 rest days, 6 working days and 3 rest days, or no fixed cycle. As shown in the report, there is an evident heterogeneity in the shift workers’ management practices used by the 10 different European ANSPs. Practices vary depending on the different characteristics of shift work, such as the direction of rotation, the duration of shifts, the organization of work individually or in teams, and the distribution of rest days. Differences can be attributed to some extent to factors such as airspace size, culture, or specific national legislation. It is not surprising, therefore, that such differences can also be observed among the extra-European providers considered in this study: Airways New Zealand (New Zealand), Air Services Australia, and the US Federal Aviation Administration (USA). In this case, the adopted rosters present are the 3/3 (Air Services Australia), the 4/2 (Air Services Australia and Airways New Zealand), the 5/2 (USA), and the 6/3 (Airways New Zealand).

### 3.2. Shift Duration

Shift duration is a factor that has been studied by several researchers, both in laboratory and in various work settings (e.g., mining, computing, and air traffic control). The durations typically considered vary between 8 and 12 h. Twelve-hour shifts have been shown to have several advantages, including allowing more days off during the week [17,25] and giving more significant opportunities for rest. Some studies also report less absenteeism [37,38] and greater job satisfaction, probably related to the possibility of having more free time [26,39,40]. Furthermore, a 12-h rostering involves fewer handovers, considered a risky time in different work contexts.

Twelve-hour shifts, however, also have disadvantages. Some studies have found increased fatigue in 12-h shifts [30] and decreased performance [41,42,43]. In a review conducted on an initial sample of over five hundred articles, an adverse effect of 12-h shifts on performance was reported, compared to 8-h shifts. This effect is estimated to result in a 100% increased risk of incidents, and is particularly relevant in the case of night shifts, as demonstrated in a study by Rosa and Colligan [44]. Several other studies have confirmed this effect [45,46,47,48]. Another example is provided by Goode [49], who found that even when the duration of the shift is between 10 and 12 h, the incident rate is slightly higher than with a duration of fewer than 8 h. His study included pilots, and the author related the frequency of incidents in operation to the hours of activity.

When choosing between a 12-h shift and an 8-h shift, another aspect must also be considered: while there is no evidence that having more time to rest is sufficient for a full recovery, on the contrary, the long-term adverse effects of fatigue accumulation are well documented [37,50]. In light of these problems, Rosa [32] suggests using shifts lasting more than 8 h only in particular working contexts and after taking a series of measures related to the frequency and duration of breaks. The research conducted by Schroeder, Rosa, and Witt [51] on behalf of the Civil Aerospace Medical Institute (CAMI) specifically concerned ATCOs. The research aimed to compare the effects of 8-h and 10-h shifts on both the vigilance and test performance of ATCOs. Fifty-two highly experienced ATCOs were involved in the research: half of the participants carried out the 10-h program, and the other half carried out the 8-h program (type 2-2-1 rotation: the first two afternoon shifts followed by two morning shifts and, later, a night shift). In the study, the authors used a modified version of the National Institute of Occupational Safety and Health (NIOSH) Fatigue Test Battery to measure eight variables: the quantity of sleep; quality of sleep; mood; physical diseases; perceived workload (indicated by the Task Load Index); response times; arithmetic task performance; and grammar reasoning [51]. The researchers collected these performance data three times during the shift: at the beginning, two hours before the completion of the shift, and at the end of the shift. The results of the study showed an increase in reaction times for both 8-h shifts and 10-h shifts during morning shifts. However, there were no differences between 8-h and 10-h shifts in performance during the day or afternoon shift in the first 4 days of rotation. Additionally, the researchers observed a decrease in performance on the last day of the 8-h rotation, corresponding to the night shift. Data on the amount of sleep reported by controllers were consistent with previous studies. In the first 4 days of rotation, there were no differences between 8-h and 10-h shifts. The maximum hours of sleep were reported on the night before the first day of rotation of the shift (8.3 h), while the minimum number of hours of sleep (5.75 h) was reported earlier on the fourth day. In both groups, the reported sleep hours decreased during the week, to a minimum of 3.75 h before the night shift in the 8-h program.

### 3.3. Rest Period between Consecutive Shifts Cycles

A review published by Fischer, Lombardi, Folkard, Willetts, and Christiani [52] considered five studies analyzing the risk of incidents as a function of the number of consecutive working days. The authors analyzed the data reported in these studies and obtained a risk index based on the number of consecutive days of service. The risk of incidents increased by about 2% on the second day, by 7% on the third day, and by 17% on the fourth-day shift compared to the first day. These results indicate the existence of an increased risk in subsequent day shifts. However, the same study shows that the risk is higher in the presence of a succession of night shifts. In an extensive study aimed at defining a Fatigue Index, Spencer, Robertson, and Folkard [53] suggested limiting the maximum number of consecutive working days to no more than six and providing for a minimum of two consecutive rest days.

Despite the relevance of this issue, few studies have considered the distribution of rest periods within a cycle of irregular shifts. Below, we present several studies conducted on specific categories of workers other than ATCOs. Interesting results are provided by the work of Folkard and Tucker [54], who conducted a questionnaire-based study involving British aircraft maintenance engineers. The study aimed to provide recommendations for the design of work shifts for staff, linking working hours (e.g., weekly worked hours, shift duration, breaks, annual leave, and notice days) and performance measures, including guidance on vigilance, the likelihood of making mistakes, and confidence in driving home after working hours. In light of their results, the authors recommended a limit of seven consecutive working days before a break of at least 2 days off between two shift cycles.

Åkerstedt and colleagues [21] sought to gather the information that would be useful for defining an optimal balance of working and rest days, in the absence of information presented in the scientific literature. The authors then analyzed data from previous studies on workers in different sectors who were on irregular shifts, or for whom the rest periods and the regularity of shifts were not constant. Subjects were investigated using the Karolinska Sleepiness Scale [55], a self-report measure of sleepiness level on a 9-point scale (9 = the maximum level of drowsiness, and 1 = the maximum level of alertness) validated against electroencephalogram (EEG) parameters. The workers were involved in different kinds of schedules: irregular shift patterns (locomotive train); traditional three-shift work (workers in the chemical industry); 12-h day and night shifts (workers in the chemical industry); rapidly rotating shift system (paper industry); weekly shifts of 84 h (construction workers who have had seven-day shifts in a row of 12 h each between 7.00 and 19.00, followed by a week of vacation); two weeks of consecutive 12-h night shifts followed by three weeks of vacation (workers on an oil production platform in the North Sea from 19:00 to 7:00); irregular and jet-lagged shifts (cabin crews); and a control group (daily shifts, 5 days of work and 2 days off). The data provided by these studies led Åkerstedt and collaborators [21] to conclude that 2 days of recovery appear to be needed after periods involving a sequence of long working hours.

A study by Totterdell and colleagues [29] also sought to identify the time needed to recover energy after a shift cycle. The study compared the levels of activation, pleasantness, tranquility, social and work satisfaction, mental workload, and reaction times in memory tasks, for 1 or 2 days of rest after 3 days of service. For some variables, including mood, job satisfaction, and performance in memory tasks, the results did not find significant differences. In contrast, 2 days of rest were associated with better values of activation, mood, social satisfaction, and mental workload than a single day of rest. In conclusion, 24 h does not seem to be sufficient time for a full recovery, even after a cycle of three service periods. With a similar goal, Rosa and Colligan [39] showed that 2 days of rest are enough to normalize most psychological functions after a 60-h working week. These results indicate the need for a break of at least 48 h following a shift cycle of approximately 60 h.

### 3.4. Rest Period between Consecutive Shifts Cycles including Night Hours

A further variable to consider is the presence of shifts that include night hours, with consequent accumulation of fatigue due to changes in biological rhythms. Knauth, Rutenfranz, Herrmann, and Poeppl [56] performed an experimental study by measuring the rhythm of body temperature following several night shifts. The results indicated that when the person was engaged in night shifts for 2 consecutive periods of service, 2 days of rest were required to restore physiological values, but after 21 consecutive nights, 3 to 4 days were necessary for the restoration of body temperature. A subsequent review by Kecklund and Akerstedt [57] cited Kecklund and colleagues [58] reporting that 3 days were needed to recover energy following a cycle of night shifts.

A study by Cruz and Rocco [33] focused on ATCOs, comparing the sleep quality of a group of ATCOs in three different shift programs, two based on a fast and counter-clockwise rotation, and one fixed with the start of the shift in the morning. One of the counterclockwise rotation programs was 2-2-1, with the first two afternoon shifts followed by two morning shifts and, later, a night shift. The minimum duration of the service periods was 8 h. The second roster with quick rotation, instead, consisted of two afternoon shifts followed by a mid-day shift and two morning shifts (2-1-2 type roster). In this case, there were two 12-h shifts and no night shift. The variables analyzed were sleep and wakefulness times, total sleep time, and subjective assessments of sleep quality and drowsiness. The results showed that the roster with the night shift was associated with fewer hours of sleep. The average number of hours of sleep decreased from about 8 h before the afternoon shifts to 5 h before the morning shifts, and to just 2.4 h before the night shift [33]. The results of this study also confirmed what had been previously found [33,51,59], although in these cases, the minimum sleep hours were higher (about 3.5). In addition, in the other roster with fast rotation, there was a reduction in sleep hours, which remained substantially more significant than in the first shift system. In that case, the amount of rest decreased from 8 h of sleep before the first 3 shifts (the two afternoons and the day shift) to 6 h before the last two morning shifts. In this case, there was a substantial difference between the hours of sleep, even where there were no consecutive periods of service that include night hours.

The reduction in sleep hours in conjunction with a service period that includes night hours increases when a roster provides for multiple consecutive night shifts. A study by Härmä and collaborators [60] investigated the relationship between periods of service that include night hours and perceived fatigue during work and on days off. Using a questionnaire, the authors collected information on a sample of more than 7000 hospital employees. Surveys took place at different stages of collection between 2008 and 2015, and the data were associated with daily records of working time in the 3 months preceding each survey. The authors found an association between the performance of several periods of service with night hours and fatigue level (both during work and on days off), altered sleep duration, and difficulty falling asleep. The suggestion resulting from the research was to limit the allocation of consecutive night shifts as much as possible.

The same conclusion was reached by Folkard and Tucker [54] in a review on the same subject. The authors summarized data on the proportion of incidents as a function of various shift characteristics, including the presence of several consecutive night shifts. The studies considered in the review investigated the relationship between incidents and the number of consecutive shifts, night shift time, and consecutive service periods, including night hours. Concerning the latter variable, data on the number of incidents were summed up between seven different studies and subsequently expressed in proportion regarding the first night shift. Authors found that the average risk increased by about 6% between the first and second night, by 17% on the third night, and by 36% on the fourth night.

Despite a moderately pronounced effect, the authors report the absence of data useful to explain this phenomenon. Indeed, the studies analyzed showed a clear increasing trend in the relative risk of incidents following consecutive night shifts, but there is insufficient information to explain this effect. A possible interpretation lies in the loss of sleep associated with the performance of a service that includes night hours, which accumulates when multiple shifts are worked out at night in succession, without the availability of a night to be able to recover from sleep loss. In the same study and using the same methodology, the authors also compared four consecutive daily shifts to determine whether the increased risk was due exclusively to the night shift or, in general, to the succession of shifts. The results showed a slight increase after four consecutive day shifts. The proportion of the incident risk was approximately 2% more on the second day, 7% more on the third day, and 17% more on the fourth-day shift than on the first day.

The comparison of these studies demonstrate that the increase in risk of incidents is greater after consecutive night shifts than when subsequent shifts are daytime. An additional risk in performing consecutive night shifts is the so-called “night shift paralysis”, reported by several ATCOs and described as a short-lived but disabling paralysis that occurs during the night shift, when workers must maintain a state of wakefulness despite the pressure of sleep. This phenomenon was reported by 6% of a group of 435 ATCOs in a survey conducted by Folkard and Condon [61] and was related to sleep deprivation.

### 3.5. Minimum Rest Periods following a Day Shift

The results reported so far refer to the rest period available following consecutive cycles of shifts, whether day or night. However, the accumulation of fatigue also depends on the amount of rest available between individual shifts, understood as the time between the end of one shift and the beginning of the next. This interval is typically variable, and its reduction typically results in a corresponding reduction in the duration of sleep. One way to allow the operator to avoid an accumulation of sleep loss is to identify the interval between two shifts necessary for sufficient rest.

A series of studies commissioned by CAMI in 1999 collected information on this issue involving ATCOs. The objective of the research program was to monitor sleep, mood, fatigue, and cognitive performance in a group of controllers to assess the association between these factors and the shift system. As reported by Nealley and Gawron [9], one of these projects consisted of a study involving a 21-day data collection on controllers conducted at a Terminal Radar Approach Control (TRACON) and an Air Route Traffic Control Center (ARTCC). The objective of the study was to assess the effect of shift times and time off between shifts on quality of sleep, mood, fatigue, and cognitive performance inf ATCOs. Both self-reported scales—the Positive and Negative Affect Schedule (PANAS) and the Stanford Sleepiness Scale (SSS)—as well as objective measures (logfile), were collected over the period of 21 days. Results showed a linear relationship between quantity and quality of sleep, highlighting an overall reduction in sleep hours when the interval between two shifts decreased. For example, controllers who had 9 h of rest reported higher scores in terms of affect and sleep quality than controllers who had 8 h of rest. These and other studies confirm the importance of the amount of rest time for the maintenance of adequate performance. It seems that sleeping fewer than 7 or 8 h per night can compromise the ability to maintain prolonged attention on a task [62,63], even if only 2 h of sleep are lost [35], and a continuous and prolonged restriction over time can affect cognitive performance [64].

A shift interval of fewer than 11 h is also called a *quick return*. In a review on the topic, Vedaa and colleagues [65] found a reduction in sleep hours to 6.5 h (or fewer) following a quick return. Härmä and colleagues [60] reported the association between a change in the rate of rapid returns and the fatigue reported during the following workdays and days off, as well as an increase in difficulty falling asleep. Other studies have found associations between quick returns and increased sleepiness [24,66,67] and increased fatigue [24,68,69]. Two studies found that reducing the number of 9-h rapid returns between evening and morning shifts improved sleep and alertness [70] and caused less fatigue than a control group [71]. The study by Costa and colleagues [23] also reported a higher level of drowsiness in an 8-h shift interval compared to a 12-h interval.

Roach, Reid, and Dawson [72], on the other hand, conducted a study to identify the minimum number of hours of break needed for people to sleep for at least 6 h between two shifts. The data refer to a sample of train drivers who had 24-h shifts. The authors reported the results in terms of hours of sleep as a function of the duration of the interval, which could be 12, 16, or 24 h, and of the start of the interval within 24 h. The results showed that participants slept, on average, 5.2 h when they had a 12 h break, from a minimum of 3.1 h for the break that began in the morning (between 08:00 and 10:00) to a maximum of 7.9 h for the break that began in the evening (between 20:00 and 22:00). In comparison, an average of 6.5 h of sleep was obtained in the 16-h intervals, again from a minimum of 4.8 h for breaks beginning in the morning (between 04: 00 and 06: 00) to a maximum of 7.7 h for breaks beginning in the evening (between 18:00 and 20:00). The average sleep time during the 24-h intervals was 8.9, from a minimum of 6.8 h for breaks beginning in the time slot between 14:00 and 16:00 to a maximum of 12.3 h for breaks beginning in the morning (between 06:00 and 08:00).

### 3.6. Minimum Rest Periods after a Period of Service That Includes Night Hours

The tendency for individuals to engage in domestic, family, or leisure activities during the day increases their sleep loss after a night shift. This aspect directly refers to the personal well-being that arises from the balance between time spent at work and time spent with family and friends (social life). A report published by EUROCONTROL [73] recommended considering the increment of ATCOs’ social needs during their career. In this regard, it is crucial for staff to be able to create/maintain an acceptable balance between work and private life. Another critical element in the definition of recovery times is the influence of biological rhythms. Recovery of sleep during the day does not have the same effectiveness as nighttime sleep, and for this reason, it is appropriate to schedule an adequate rest period following a night shift. A service period that includes night hours reduces the number of rest hours available to the operator.

It is worth pointing out that the opportunity to recover sleep hours also has a utility in a preventive perspective since prolonged sleep limitation can have effects on the brain that can continue to affect alertness and performance for days or weeks [74]. Despite this awareness, it is unclear how much time is needed to recover from the harmful effects of lack of sleep. In this regard, a report published by IFATCA, ICAO, and CANSO [18] stated how laboratory studies have yet to provide a clear answer to this question.

The EUROCONTROL study [73] suggested that a minimum time of rest after a night shift is 24 h, or preferably 48 h. Additionally, the guidelines suggested by Dinges and colleagues [75], referring to more heterogeneous work contexts, suggested planning two consecutive nights of rest to recover from sleep loss and avoid adverse effects on the level of attention. The authors proposed a standard off-duty recovery period of 36 continuous hours, to include two consecutive nights of recovery sleep, in 7 days. A study by Tucker, Smith, Macdonald, and Folkard [34] also found positive effects of having 2 days off before two night shifts. In the study, two shift patterns were compared, and the operator’s level of well-being (sleep quality, activation level, and fatigue) was investigated. The authors showed that system workers with rest periods of at least 24 h between shifts reported slightly higher average levels of attention and slightly lower levels of chronic fatigue compared to system workers without such rest periods.

One feature that determines the minimum duration of rest is the ability to fall asleep and recover from sleep loss. In this regard, the study by Härmä and colleagues [60] examined whether changes in working shifts and the intensity of working shifts were related to differences in difficulty falling asleep, fatigue, and sleep duration. The study analyzed data from hospital employees engaged in 24-h shift systems. The results showed that those who worked two consecutive night shifts reported a higher level of fatigue during work and on free days, as well as greater difficulty in falling asleep. On the contrary, the effect was not present with evening shifts.

Åkerstedt and colleagues [21] produced other data on this topic. In this case, the authors analyzed data from workers engaged in irregular shifts to check the effect of the roster on the level of drowsiness. The workers surveyed in this study complied with rosters that were only partially similar to those of an ATCO and, in many cases, performed a very long series of night shifts. Although the study was not limited to the ATM context, the information reported refers to irregular shifts based on circadian continuity, and the results are consistent with the indications reported so far. The levels of drowsiness for different groups of workers were compared using subjective data and, in some cases, sleep diaries. Following an overall analysis of these data, the authors concluded that a rest period of more than 24 h is necessary if the roster includes multiple-night shifts, that is, if it leads to a significant loss of sleep hours during the week.

### 3.7. Breaks during the Shift: How Long to Work without a Break

In many cases, the information available in the scientific literature on this topic appears to be contradictory. A possible explanation is due to the different nature of the work, as suggested by Tucker [76]. Folkard and colleagues conducted an intriguing synthesis in their work, proposing a risk index for contexts with continuous shift work. The index is obtained by applying an algorithm that allows researchers to consider various features of the shift program [52,77]. The algorithm’s features consider shift type (e.g., morning, afternoon/evening, and night shift); number of consecutive turns (for example, four night shifts consecutively in a row, from Monday evening to Friday morning); shift duration (e.g., shifts of 8 h vs. 12 h); pause intervals (e.g., minutes since last break); and the length of breaks. The risk index derives from the relationship between the roster’s features and incident frequency (or near misses). Folkard, Robertson, and Spencer [8] (p. 179) reported that: “it has been evident that there are significant differences between fatigue and risk in terms of trends shown in the characteristics of working hours.” Dababneh, Swanson, and Shell [27] carried out an experimental study on the duration and frequency of breaks. The specific interest of this study was the relationship between two break programs with employee performance and stress. One program included a break every hour, and the other included a break every half hour. Although both programs highlighted the effectiveness of the break, employees preferred when it was less frequent. In addition, a recent study [22] investigated the effect of short and frequent breaks on performance in a surveillance task (X-ray image inspection). In this case, a sample of 71 participants was divided in two experimental conditions. In one condition, participants had the option of taking a break of 10 min every 20 min, while in the other (control group) they worked continuously. The analysis conducted by the authors has highlighted the absence of differences in performance with the control group. A possible explanation for this result is that very short and frequent breaks are not necessarily useful, in line with what has been already reported by Dababneh and colleagues [27]. These results are also consistent with what was reported by Chavaillaz and colleagues [78], using a methodology similar to that described in the study by Buser and colleagues [22].

Chang, Yang, and Hsu [7] conducted a specific study on ATCOs. The authors evaluated the fatigue felt by a sample of operational controllers in the control tower of Taiwan Airport, throughout 6 day and 5 night shifts. Fatigue was assessed using the Samn-Perelli scale, a 7-point self-assessment measure. In this case, a 1-h break in daytime shifts with a duration of between 9 and 11 h was allowed and a 4.5–5-h break in night shifts of 12–14 h. The authors also considered the break’s effectiveness and the time of the day, including in their analyses breaks starting at a different moment of the shift (the beginning, the middle, or the end of the shift). The results confirmed the effectiveness of the break in all conditions, even if the beneficial effect had a relatively short duration. The fatigue values reported by the controllers were highest before the break and at the end of the shift and were lower just after the break. In this study, breaks were granted after at least 4.5 h of activity in day shifts and after at least 4 h of activity in night shifts. Table 1 shows the time slots and the duration of the breaks. 

An analysis conducted by Folkard and Tucker [54] recommended planning a break after a maximum of 4 h of activity. This information comes from a study of British aircraft maintenance engineers, in which the authors related some features of the work schedule with human performance indicators. Similar indications are also contained in the report by EUROCONTROL [73].

### 3.8. Organization of Breaks during the Service Period

The data from the literature agree with the importance of breaks to allow for a rest during the working day. However, no homogeneous indications about the rest break timing are available. There is conflicting evidence regarding the optimal duration of breaks, and it seems likely that both the optimal schedule and the beneficial effect of rest breaks depends on the work’s features [76].

Studies carried out in ATM contexts confirm that rest breaks should be adapted to the characteristics of the activity being carried out by the operator. Activities carried out by ATCOs require constant vigilance, and since performance in supervisory tasks decreases over time, a break is necessary every 2 h of continuous work [79,80]. Moreover, two studies conducted by Tucker and colleagues [81,82] found that the attention increases after taking a break only for a limited duration and only covers the first half-hour of resumption in activity. For this reason, it is essential to schedule a break of sufficient length for the recovery of energy. Especially in periods of service that include night hours, a sufficiently long break can allow the operator a short period of sleep, which is useful to recover energy. Nealley and Gawron [9] reported on the importance of a short sleep period for maintaining an adequate level of performance, pointing toa simulation study in the United States and a radar system in New Zealand. The research in the United States was a 4-day laboratory simulation involving 59 military and civilian ATCOs [83]. Physiological (EEG, EOG, EMG, and ActiGraph), performance (modified Bakan Vigilance Test), and subjective data were collected to assess whether naps have a benefit on sleepiness and performance. Both 45-min and 2-h naps were evaluated. Vigilance test scores improved after rest, but 2-h sleep produced a more critical effect on performance. However, Caldwell and Caldwell [84] reported that the side effect of the “sleep inertia” is not a good reason for restricting naps, because they produce better performance in the long run. A possible solution would be to “provide anyone who has been napping with a “grace period” of approximately 20 to 30 min to become fully awake before expecting them to handle any type of demanding mental task” (pp. 127–128).

The results suggest the validity of short sleep to improve performance, especially during night shifts. In this case, the duration should be at least 2 h over an 8-h night shift. The New Zealand study was an 8-month field study [28]. Physiological data (PSG; ActiGraph), performance data (Psychomotor Vigilance Task; PVT), and subjective indications (sleep diaries) were collected on 28 controllers during night shifts beginning late in the evening (22:30–06:00 or 23:30–06:30). The study aimed to assess the effectiveness of a 40-min nap on performance and vigilance on a night shift. As part of this assessment, the ability of controllers to sleep during the break was also considered. The average amount of sleep obtained was 19 min in the shift starting at 22:30 and 20 min in the shift starting at 23:30. No rapid eye movement (REM) sleep phases were recorded during the study. A nap opportunity improved performance, although the improvement did not last throughout the shift. It is probable that the amount of time did not allow for the derivation of a substantial benefit in the remaining duration of the shift. Although these results are promising for the beneficial role of breaks in night shifts, it is crucial to consider the relationship between breaks and circadian rhythms. In fact, a study by Dinges and colleagues [85] showed that a 2-h break in a depressive moment of the circadian rhythm can have adverse effects on performance. This suggests that in the definition of the start of the breaks, the controller should be given enough flexibility to adjust his or her circadian rhythm with the rest available.

During rest breaks, ATCOs should have time to move away from their working position, have a washroom break, or spend time in facilities for relaxation at work [79,80]. Several studies have reported that the activities during the break can make it more or less effective. Some studies, for example, have reported that breaks are more likely to improve an individual’s mood if they involve relieving activities (for example, naps, relaxation, and socialization), rather than work-related activities [86]. 

Another relevant aspect is the time when the break is scheduled. A study by Chang, Yang, and Hsu [7] compared the effectiveness of breaks in reducing the feeling of fatigue based on the break time. Especially during night shifts, the ability to recover energy depends on the break time as well as on its duration. Numerous studies have reported declines in performance when the operator must stay awake during the window of circadian low (WOCL), which is the time slot that includes early morning hours (from 02:00 to 06:00). In this case, a break that entirely or partially overlaps the WOCL period results in a lower perception of fatigue by the ATC. This result is also confirmed when a controller who has a more extended break must work during the WOCL period [7].

A further element to consider is the relationship between performance and changes in air traffic, which was also analyzed in a study published by Hagemann [87], who investigated the relationships between the complexity of the task and subjective and physiological indicators of attention. An important finding of this study was that there was an increase in effort and a decrease in performance in the supervisory task after 2.5–3 h of activity, indicating it as the time to be considered when planning a break in an air traffic control context.

## 4. Concluding Remarks

This review attempted to extract guidelines to design a shift schedule for ATCOs. The aim was to provide appropriate advice in choosing parameters for the definition of rest periods. The results were presented following a descriptive logic of the main parameters emerging from the literature. The interrelation of these parameters does not allow for the creation of a list with clear directions. Rather, it is plausible to provide indications that may justify the choices in constructing a schedule of shifts. An example is the maximum duration of a shift, which could vary between 7 h and 10 h. The indication is to stay within this range, but the choice depends on other parameters of the schedule desired for use. A longer rotation, for example, of 6 days, may involve shorter shifts, with a broader interval between two shifts and several little breaks within the shift. On the contrary, a more compressed schedule will provide longer shift duration, with a shorter interval between two shifts, but a wider gap between two programs and, of course, a different distribution of breaks within the shift. Shifts that include night hours have more significant implications for the operator’s perceived fatigue as a result of the loss of sleep that occurs both during the shift and during the rest of the day due to the predisposition toward wakefulness during the daytime hours [16]. Based on the information identified in the literature, it is appropriate to plan a good recovery after a night shift [56,57,60] and, where possible, avoid scheduling consecutive service periods, including night hours. Opportunities for rest must be adequate for the time of the day in which the shift takes place, and this applies to both the interval between two shifts and the break during the shift. The recommendation is to provide more extended and more frequent rest periods after a night shift compared to a day shift. Numerous studies have reported a more pronounced decline at the end of a night shift [88,89,90,91]. This decline could be due to several factors, including fatigue, drowsiness, sleep deprivation, and low task load [92,93,94,95,96,97]. Consequently, there should be a break of higher duration and higher frequency in night shifts. During a day shift, conversely, a proportion of break time to shift time of about 20–25%, corresponding to approximately a half-hour break every 2 h of work, should be sufficient (Table 2 and Table 3) [7,79,80].

Fatigue in air traffic management is an inevitable hazard and ANSPs are keen to address its safety implications. ICAO (e.g., Annex 11, Appendix 7) and EASA (see the EC 217/373) recommend the development and implementation of a Fatigue Risk Management System in recognition of the issue’s relevance, and to provide the ANSPs with guidelines and policies to mitigate the fatigue risk.

In this framework, future research on professional fatigue may play a significant role, supporting the ATS providers in defining a proactive evidence-based approach to fatigue management.

Particularly, sleep science may contribute to the development of an integrated approach to fatigue: identifying evidence to improve physiological and behavioral knowledge on how to recover from sleep loss (across one night or multiple nights of shift) or how the circadian body clock affects the timing and quality of sleep (and then performance). 

Moreover, research on fatigue is expected to also contribute to support the ATS providers in developing reliable tools for monitoring the fatigue of ATCOs, by considering relevant indicators.

Currently, the way to manage fatigue in the European ANSPs remains fragmented and the monitoring approaches implemented are quite different across ANPSs. Research efforts in this field may contribute to the harmonization process of fatigue management.

This review does not discuss the implications of recent technological ATM innovations. The Remote Tower Center (RTC), for example, requires an air traffic controller to work in “multiple positions”, which means handling two or more airports from one remote tower module. The maximum number of airports assigned to one controller, the maximum number of movements per controller, and the maximum number of controllers per airport are only a few examples of the resulting implication of designing an efficient roster plan in an RTC. However, research is ongoing and this novelty has not yet been fully addressed in the literature. Future reviews should also approach this major change.

In conclusion, this work represents an attempt to provide directly transferable information in defining shift schedules for ATCOs. The indications identified have made it possible to partially achieve the objective of the work. The suggested parameters can help construct shift schedules within a variation margin that depends on individual application contexts. The definition of specific parameters remains somewhat abstract and open to interpretation, thus representing a work limitation.

## Figures and Tables

**Table 1:** Work schedule in day shifts (DS) and in night shift (NS).

Shift	Work Hours	Break Time	Total Break Time
DS 1	07:30–18:30	13:00–14:00	1 h
DS 2	07:30–18:30	13:00–14:00	1 h
DS 3	07:30–18:30	13:30–14:30	1 h
DS 4	07:30–17:30	12:00–13:00	1 h
DS 5	08:00–18:00	12:30–13:30	1 h
DS 6	08:00–17:00	14:00–15:00	1 h
NS 1	19:00–08:00	23:30–04:30	5 h
NS 2	18:00–06:00	23:00–02:00, 04:30–06:00	4.5 h
NS 3	18:00–08:00	03:00–08:00	5 h
NS 4	18:30–08:30	02:00–07:00	5 h
NS 5	18:00–08:00	22:00–03:00	5 h

Adapted with permission from Chang, Yang, and Hsu, 2019.

**Table 2:** Indications on the minimum number of days of rest between consecutive shift cycles.

Rest Period between Consecutive Shifts	Reference
2 days off after 6 days of service	Fischer and colleagues (2017)
7 working days and 2 days off between 2 shift cycles	Folkard and Tucker (2003)
2 days off after periods involving a sequence of long working hours	Åkerstedt and colleagues (2000)
2 days off after 3 days of service	Totterdell and colleagues (1995)
2 days of rest after a 60 h working week	Rosa and Colligan (1988)

**Table 3:** Rostering features and reported durations.

Rostering Features	
Shift duration	8 ÷ 12 h
Rest period between consecutive shifts	2 days off after 3/7 days of service
Rest period between consecutive shifts including night hours	2 days off after 2 consecutive periods of service including night hours 4 days off after an extended period of service including night hours
Minimum rest periods following a day shift	11 ÷ 12 h [65,72]
Minimum rest periods after a period of service that includes night hours	24 ÷ 36 h [73,75]
Breaks during the shift: How long to work without a break	Day shift 4 ÷ 4.5 h [7,54] Night shift 4 h [7]
Organization of breaks during the service period	2 ÷ 3 h [84,87]
Minimum rest periods after a period of service that includes night hours	24 ÷ 36 h [73,75]
